# A novel prognostic signature for lung adenocarcinoma based on cuproptosis-related lncRNAs: A Review

**DOI:** 10.1097/MD.0000000000031924

**Published:** 2022-12-09

**Authors:** Huang Di, Jiting Zhao, Xue Zhu, Xinpeng Zhou, Yuanlong Hu, Mengjie Wang, Zhanjun Qiu, Wei Zhang, Xianhai Chen

**Affiliations:** a First Clinical Medical College, Shandong University of Traditional Chinese Medicine, Jinan, China; b Department of Respiratory and Critical Care Medicine, The Affiliated Hospital of Shandong University of Traditional Chinese Medicine, Jinan, China; c Department of Gastroenterology, The Affiliated Hospital of Shandong University of Traditional Chinese Medicine, Jinan, China; d Department of Rheumatology, The Affiliated Hospital of Shandong University of Traditional Chinese Medicine, Jinan, China.

**Keywords:** cuproptosis, cuproptosis-related lncRNAs (CRlncRNAs), lung adenocarcinoma (LUAD), TCGA

## Abstract

Lung adenocarcinoma (LUAD) is a highly heterogeneous disease with complex pathogenesis, high mortality, and poor prognosis. Cuproptosis is a new type of programmed cell death triggered by copper accumulation that may play an important role in cancer. LncRNAs are becoming valuable prognostic factors in cancer patients. The effect of cuproptosis-related lncRNAs (CRlncRNAs) on LUAD has not been clarified. Based on the Cancer Genome Atlas database, CRlncRNAs were screened by co-expression analysis of cuproptosis- related genes and lncRNAs. Using CRlncRNAs, Cox and LASSO regression analyses constructed a risk prognostic model. The predictive efficacy of the model was assessed and validated using survival analysis, receiver operating characteristic curve, univariate and multifactor Cox regression analysis, and principal component analysis. A nomogram was constructed and calibration curves were applied to enhance the predictive efficacy of the model. Tumor Mutational Burden analysis and chemotherapeutic drug sensitivity prediction were performed to assess the clinical feasibility of the risk model. The novel prognostic signature consisted of 5 potentially high-risk CRlncRNAs, MAP3K20-AS1, CRIM1-DT, AC006213.3, AC008035.1, and NR2F2-AS1, and 5 potentially protective CRlncRNAs, AC090948.1, AL356481.1, AC011477.2, AL031600.2, and AC026355.2, which had accurate and robust predictive power for LUAD patients. Collectively, the novel prognostic signature constructed based on CRlncRNAs can effectively assess and predict the prognosis of patients and provide a new perspective for the diagnosis and treatment of LUAD.

## 1. Introduction

Lung cancer (LC) has a high morbidity and mortality rate and poor prognosis, resulting in a heavy disease burden, with an estimated 2 million new cases and 17.6 million deaths per year.^[1]^ Lung adenocarcinoma (LUAD) is a common subtype of LC that develops from small airway epithelial cells and type II alveolar cells and accounts for approximately 40% of all LC.^[2,3]^ Despite many advances in diagnostic and therapeutic strategies for LUAD, it remains 1 of the most aggressive and rapidly lethal tumor types, with a low early diagnosis rate, high late mortality, and poor prognosis. Therefore, there is an urgent need to explore more complete clinical diagnostic methods for LUAD and to discover new and effective biomarkers and therapeutic targets to accurately predict and improve the clinical prognosis of patients with LUAD.

Long non-coding RNAs (lncRNAs) are a series of non-coding transcripts over 200 nucleotides in length that do not encode proteins but play an important role in oncological diseases.^[4,5]^ Related studies have shown that aberrantly expressed lncRNAs have an important prognostic value in tumor diseases.^[6–8]^ Cuproptosis, a form of programmed cell death caused by intracellular copper accumulation that triggers the aggregation of mitochondrial lipidated proteins and the destabilization of Fe-S cluster proteins, may be a promising strategy for tumor treatment.^[9,10]^ At present, little is known about the potential mechanisms of copper death in LUAD, and studies of Cuproptosis-related lncRNAs (CRlncRNAs) in LUAD are yet to be systematically explored. Therefore, the identification of CRlncRNAs closely related to the prognosis of LUAD and the exploration of potential targets related to the mechanism of copper death in LUAD are of great importance for the study of the mechanism of LUAD, as well as the diagnosis, prognosis, and clinical treatment of the disease.

Based on the LUAD dataset from The Cancer Genome Atlas (TCGA), we screened 10 CRlncRNAs to construct a new prognostic model for LUAD, aiming to improve the clinical diagnostic and prognostic accuracy of LUAD and discover potential biomarkers and therapeutic targets, to open new perspectives for individualized precision diagnosis and treatment.

## 2. Methods

### 2.1. Data sources and processing

LUAD RNA-seq transcriptomic, clinical, and tumor mutational burden (TMB) data were downloaded from TCGA database (https://portal.gdc.cancer.gov/). RNA-seq transcriptome expression data were normalized and missing samples in the clinical data profile were excluded. Eighteen Cuproptosis-related genes (CRGs) were retrieved from literature,^[9,11–15]^ including NLRP3, ATP7B, ATP7A, SLC31A1, FDX1, LIAS, LIPT1, LIPT2, DLD, DLAT, PDHA1, PDHB, MTF1, GLS, CDKN2A, DBT, GCSH, and DLST. The LUAD transcriptome data were collated, the Perl (Strawberry Perl 5.30) script was used to distinguish between mRNAs and lncRNAs, and the R language (Rx64 4.1.0) “limma” package was used to extract the expression of CRGs. The expression files of the CRGs and lncRNAs were analyzed using Cor. The test function and Wilcoxon test for co-expression and filtering conditions (cor > 0.4, *P* < .001) were used to filter out CRlncRNAs. All data in this study were obtained from public databases and did not involve informed consent was not obtained from the patients involved.

### 2.2. Construction and assessment of PRlncRNAs prognostic signature for LUAD

To construct the best LUAD PRlncRNA prediction prognostic model, tumor samples were randomly divided into training and test cohorts at a 1:1 ratio, and the chi-square test was applied to compare the differences between groups. The training cohort data were used for prognostic model construction, and the test cohort and the total cohort were used for model validation. Using R “survival,” “ survminer” and “glmnet” packages, we first identified potential crlncrnas by univariate Cox regression analysis (*P* < .01), then reduced overfitting genes by least absolute shrinkage and selection operator (LASSO), and finally constructed the best risk prognosis model through the results of multivariate Cox regression analysis. The formula for calculating the prognostic risk score of the PRlncRNAs for each LUAD sample was as follows:


$$\begin{array}{l} Risk\;Score=\sum_{i=1}^{n}\left(expr_{i}\times coef_{i}\right) \end{array}$$

where *expr*_i_ represents the expression of each lncRNA and coef_i_ represents the corresponding coefficient. According to the median risk scores, LUAD samples were divided into high- and low-risk groups. The expression of PRlncRNAs involved in risk model construction was extracted and correlated with CRGs to explore the potential mutual regulatory relationships between them.

The Kaplan–Meier survival analysis, risk scoring curves, and survival state scatterplot were applied to the training cohort using the R“survival” and “ survminer” packages to compare the survival differences between high- and low-risk groups, and the test cohort and the total cohort were applied for validation. Survival analysis was performed on the total cohort by drawing a receiver operating characteristic (ROC) curve and applying the area under the curve (AUC) to evaluate the model’s predictive performance. Using univariate and multivariate Cox regression analyses, we assessed whether the risk model can be used as an independent prognostic factor for LUAD. Clinical subgroups were established according to age (<= 65 or > 65 years), sex (male or female), and clinical stage (stage I–II or III-IV). Kaplan–Meier survival analysis was performed to verify whether the model could be used in patients with different clinical characteristics. The R “rms “^[16]^ packages were applied to construct a nomogram to predict survival according to sex, age, disease stage, and risk groups, and Calibration charts were drawn to test the accuracy of the nomogram.

### 2.3. Principal component analysis

Principal component analysis (PCA)^[17]^ was performed to compare the differences between the high- and low-risk groups based on all gene sets, the CRGs set, the CRlncRNA set, and the prognostic model 10-CRlncRNA set.

### 2.4. Tumor mutational burden analysis

TMB is the total number of non-synonymous mutations in each coding region of the tumor genome, and its calculation can indirectly reflect the ability and extent of neoantigen production by tumors.^[18,19]^ Based on the LUAD mutation data downloaded from TCGA database (category: simple nucleotide variation, type: masked somatic mutation, format:maf), TMB was calculated (TMB = Somatic/L, Somatic is the total number of mutations, and L is the size of the effective coding region). The R “maftools” package was applied to analyze the mutation data and construct a Waterfall Plot to visualize the mutations in the high-risk and low-risk groups. To investigate the prognostic value of TMB, LUAD samples were divided into high TMB (H-TMB) and low TMB (L-TMB) groups according to the median TMB score, and survival analysis was performed with a risk score to compare the differences between the groups.

### 2.5. Chemotherapeutic drugs sensitivity prediction

The half-maximal inhibitory concentration (IC50) is the amount of drug required to inhibit a biological process by half, with lower values indicating greater drug sensitivity, and is widely used as a measure of drug efficacy.^[20,21]^ The “pRRophetic” package can predict the IC50 of chemotherapeutic drugs and the sensitivity of chemotherapeutic drugs from the genetic level.^[22]^ Based on the Cancer Genome Project (CGP) cell line expression profiles and TCGA LUAD gene expression profiles, we applied the R “pRRophetic” package to predict The IC50 of sensitive chemotherapeutic drugs was screened by the Pearson correlation coefficient, and the IC50 of sensitive chemotherapeutic drugs was analyzed by Wilcoxon rank-sum test to determine the difference of IC50 between high and low-risk groups.

## 3. Results

### 3.1. Construction and assessment of PRlncRNAs prognostic signature for LUAD

**Figure 1.**
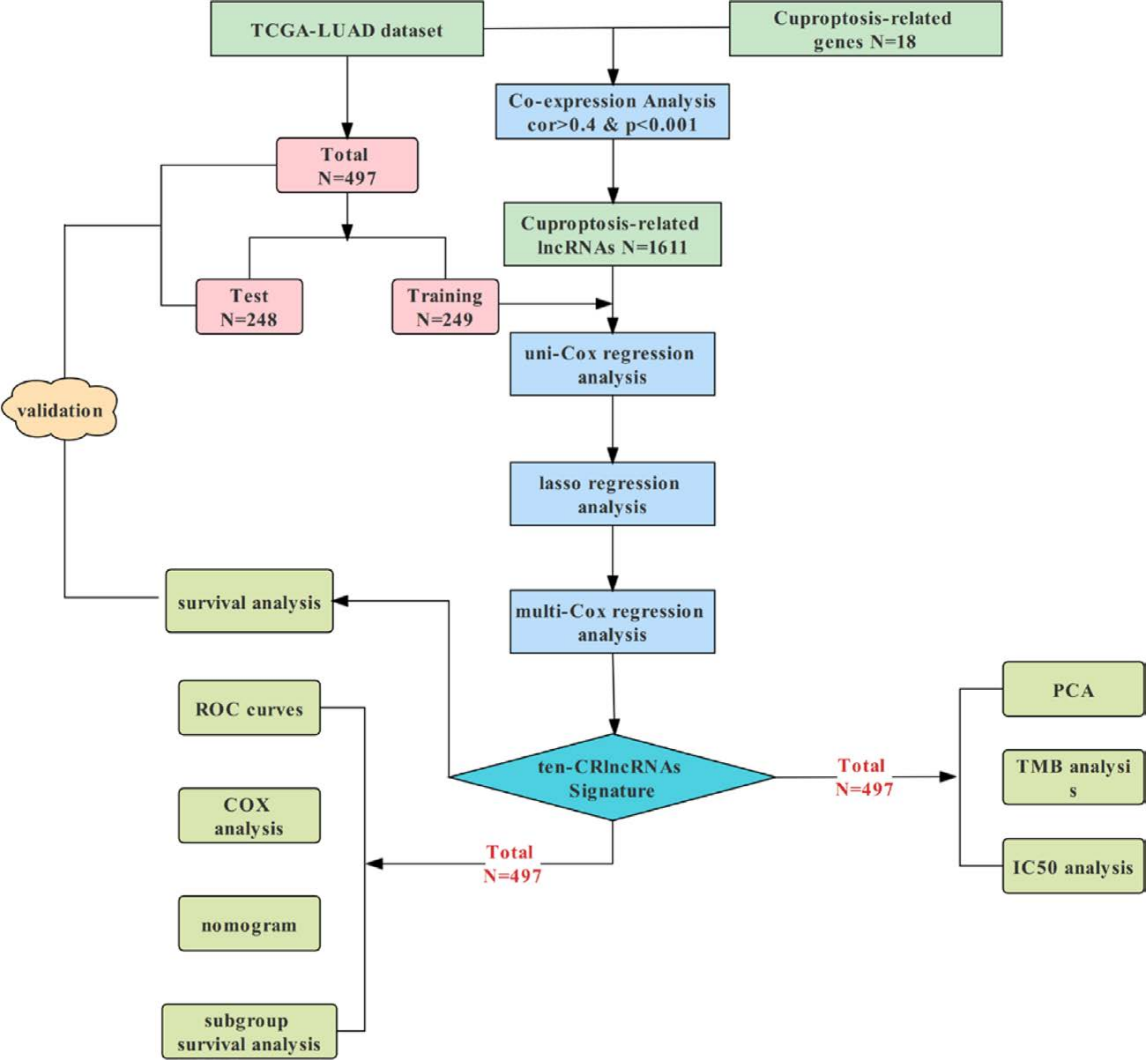
Study flowchart shows the process of constructing and assessing the 10-CRlncRNAs prognostic signature for lung adenocarcinoma. CRlncRNAs = Cuproptosis-Related lncRNAs.

**Figure 2.**
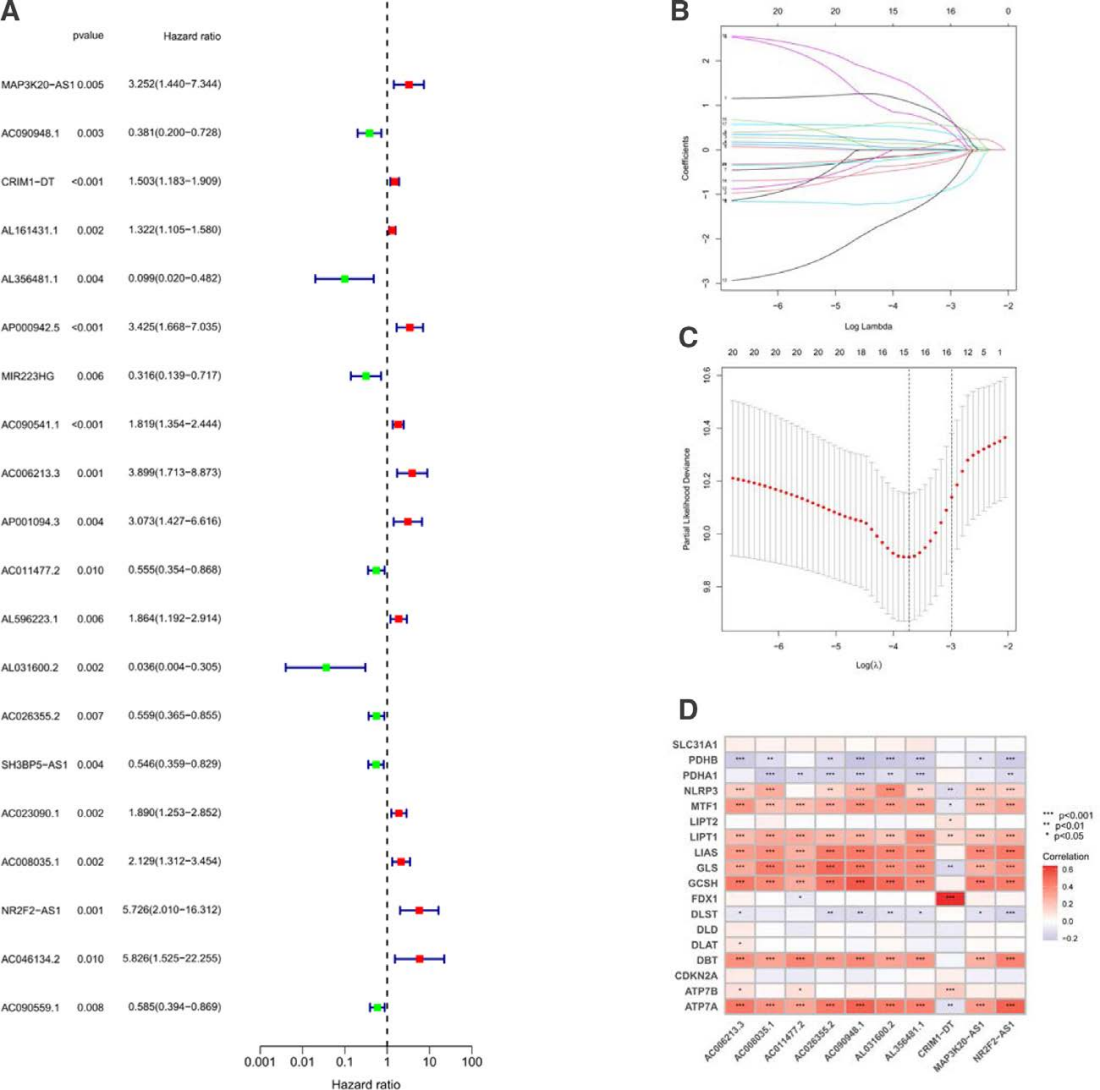
(A) Forest plot of univariate Cox regression analysis; (B) The dynamic process of applying Lasso regression analysis to filter variables; (C) Selection process of the optimal cross-validation parameter λ in the LASSO model; (D) Correlation Heatmap of CRlncRNAs with CRGs, red color indicates a positive correlation and blue color indicates negative correlation. CRGs = cuproptosis-related genes, CRlncRNAs = cuproptosis-related lncRNAs, LASSO = least absolute shrinkage and selection operator.

A flowchart for constructing and assessing the prognostic signature of PRlncRNAs in LUAD is shown in Figure 1. Based on the CRGs and CRlncRNA expression files, co-expression analysis (cor > 0.4, *P* < .001) was performed, and 1611 significantly correlated CRlncRNAs were obtained. Samples with missing expression and clinical data were removed, and the included tumor samples (n = 497) were randomly divided into a training cohort (n = 249) and a test cohort (n = 249) in a ratio of 1:1. There were no significant differences (*P* < .05) in age, sex, tumor stage, or other clinical characteristics between the groups (Table 1). The expression files of the training cohort of CRlncRNAs were subjected to univariate Cox regression analysis (*P* < .01), and 20 candidate CRlncRNAs were found to be significantly associated with survival (Fig. 2A). Overfitting genes were reduced by LASSO constraint parameters (Fig. 2B and C), and multivariate Cox regression analysis was performed to screen 10 CRlncRNAs to construct the best risk prognosis model. Risk Score = [MAP3K20-AS1 expression × (1.8070)] + [AC090948.1 expression × (−0.5232)] + [CRIM1-DT expression × (0.2365)] + [AL356481.1 expression × (−1.6709)] + [AC006213.3 expression × (0.9358)] + [AC011477.2 expression × (−0.4754)] + [AL031600.2 expression × (−2.2810)] + [AC026355.2 expression × (−0.6656)] + [AC008035.1 expression × (0.7825)] + [NR2F2-AS1 expression × (2.4483)]. ACCORDING TO THE MEDIAN RISK SCORES, the LUAD samples were divided into a high-risk group and a low-risk group. The CRGs were correlated with the 10 CRlncRNAs involved in model construction by line analysis (Fig. 2D), and the results showed that FDX1 had a highly significant positive correlation with CRIM1-DT (*P* < .001) and PDHB had a highly significant negative correlation (*P* < .001).

**Table 1:** The sociodemographic information of patients.

Characteristics	Total (n = 497)	Test (n = 248)	Training (n = 249)	*P* value
Age, n (%)				.5256
age ≤ 65	239 (48.09%)	116 (46.77%)	123 (49.40%)	
age > 65	258 (51.91%)	132 (53.23%)	126 (50.60%)	
Gender, n (%)				.1145
Female	268 (53.92%)	143 (57.66%)	125 (50.2%)	
Male	229 (46.08%)	105 (42.34%)	124 (49.8%)	
Stage, n (%)				.4831
Stage I	265 (53.32%)	128 (51.61%)	137 (55.02%)	
Stage II	118 (23.74%)	54 (21.77%)	64 (25.7%)	
Stage III	80 (16.1%)	45 (18.15%)	35 (14.06%)	
Stage IV	26 (5.23%)	14 (5.65%)	12 (4.82%)	
Unknown Stage	8 (1.61%)	7 (2.82%)	1 (0.4%)	
T, n (%)				.6653
T1	168 (33.8%)	84 (33.87%)	84 (33.73%)	
T2	262 (52.72%)	127 (51.21%)	135 (54.22%)	
T3	45 (9.05%)	23 (9.27%)	22 (8.84%)	
T4	19 (3.82%)	12 (4.84%)	7 (2.81%)	
unknown	3 (0.6%)	2 (0.81%)	1 (0.4%)	
M, n(%)				.5442
M0	328 (66%)	156 (62.9%)	172 (69.08%)	
M1	25 (5.03%)	14 (5.65%)	11 (4.42%)	
unknown M	144 (28.97%)	78 (31.45%)	66 (26.51%)	
N, n(%)				.4693
N0	320 (64.39%)	155 (62.5%)	165 (66.27%)	
N1	93 (18.71%)	47 (18.95%)	46 (18.47%)	
N2	70 (14.08%)	37 (14.92%)	33 (13.25%)	
N3	2 (0.4%)	2 (0.81%)	0 (0%)	
unknown N	12 (2.41%)	7 (2.82%)	5 (2.01%)	

**Figure 3.**
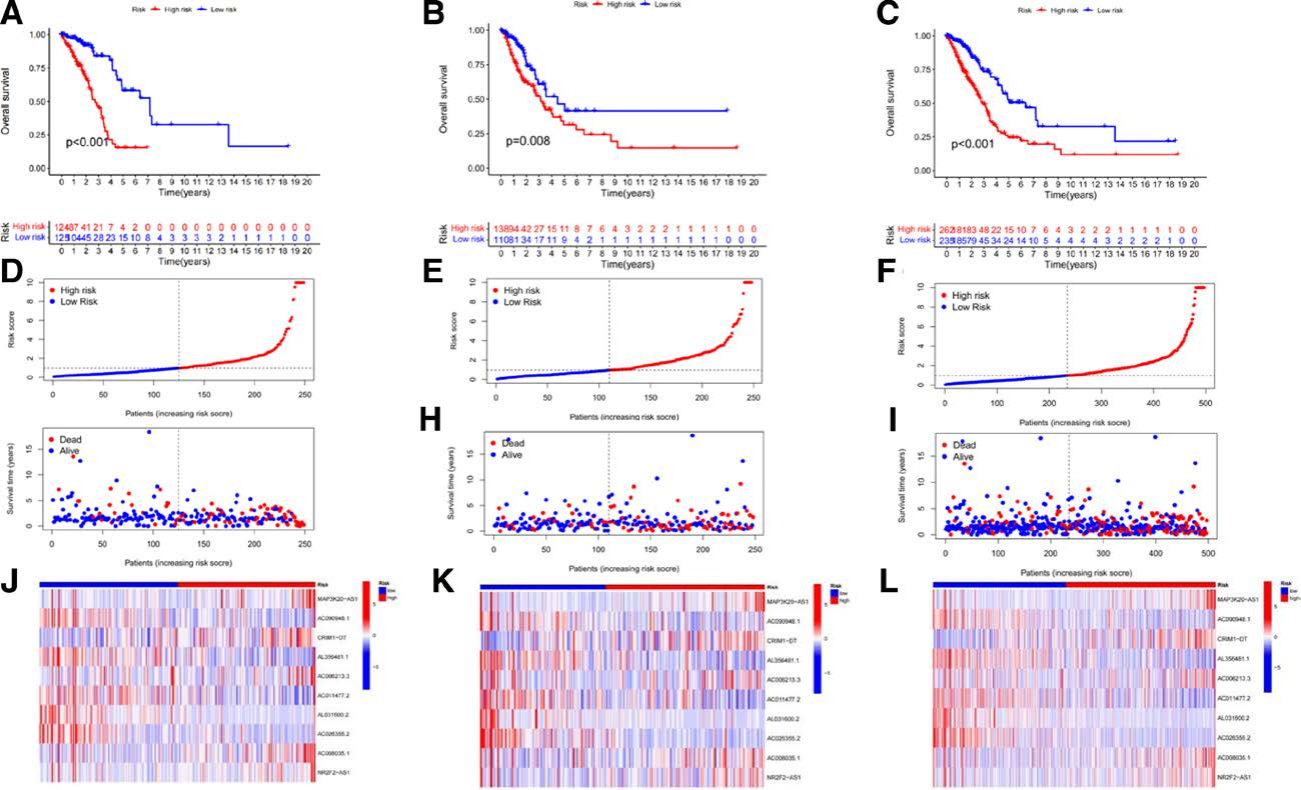
(A–C) Kaplan–Meier survival curves; (D–F) Risk Scoring Curves; (G–I) Survival state scatterplot, the green dots represent survival and the red dots represent death; (J–L) Heatmap of 10 CRlncRNAs expressions in the high-risk and low-risk groups for the construction of prognostic signature. CRlncRNAs = cuproptosis-related lncRNAs.

Kaplan–Meier analysis showed that overall survival (OS) was better in the low-risk group than in the high-risk group, suggesting that the risk score could predict OS (Fig. 3A). Risk scores and survival status were visualized using risk-scoring curves and state scatterplots. The results showed that higher risk scores resulted in higher mortality, indicating that risk scores can predict mortality (Fig. 3D and G). The expression heatmap of 10 CRlncRNAs in the model in the high- and low-risk groups (Fig. 3J) showed that AC090948.1, AL356481.1, AC011477.2, AL031600.2, and AC026355.2 were highly expressed in the low-risk group, and AP3K20-AS1, CRIM1-DT, AC006213.3, AC008035.1, and NR2F2-AS1 were highly expressed in the high-risk group. To further test the predictive performance of the model, the test cohort (Fig. 3B, E, H, and K) and the total cohort (Fig. 3C, F, I, and L) were used for validation, and the results were the same as those of the training group.

**Figure 4.**
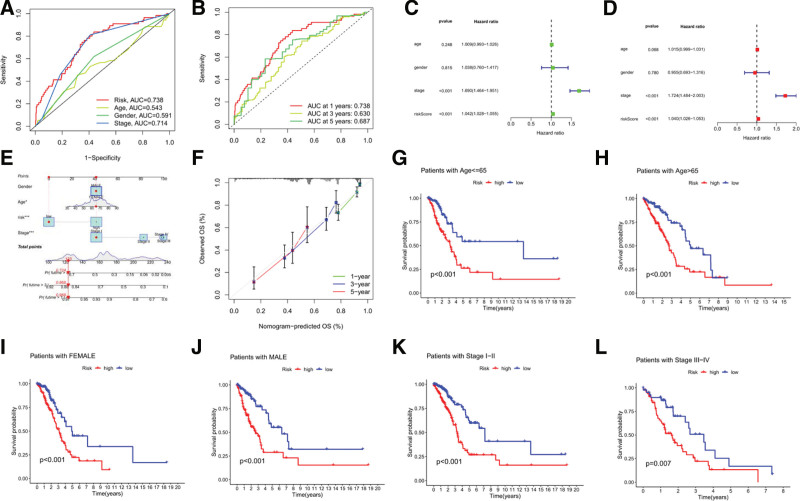
(A) Clinical Multi-index ROC curves for The AUC of risk score, age, gender, and stage of LUAD patients; (B) Time-dependent ROC curves for The AUC of overall survival at 1, 3, and 5 years of LUAD patients; (C) Univariate Cox regression analysis; (D) Multinomial Cox regression analysis; (E) Construction of a nomogram combined clinical indicators and risk score for predicting survival probabilities at 1, 3, and 5 years of LUAD patients; (F) Calibration charts for validating the predictive accuracy of the 1-year, 3-year, and 5-year survival probabilities of the nomogram. (G–L) Subgroups of clinical indicators Kaplan–Meier survival analysis of LUAD patients. (G) Age<=65; (H) Age > 65; (I) Female; (J) Male; (K) Stage I–II; (L) Stage III–IV. AUC = area under the curve, LUAD = lung adenocarcinoma, ROC = receiver operating characteristic curve.

The receiver operating characteristic (ROC) curves can evaluate the accuracy of a diagnostic test by the AUC.^[23]^ Clinical multi-index ROC curves showed an AUC value of 0.738 for the risk score, higher than other clinical factors (Fig. 4A). Time-dependent ROC curves predicted OS at 1, 3, and 5 years with AUC values of 0.738, 0.630, and 0.687 respectively (Fig. 4B). This indicates that the model has better predictive power for risk scores than for other clinical factors, and has predictive power for survival time. In the univariate Cox regression analysis, the hazard ratio of the risk score was 1.042 (95% CI 1.028-1.055) (*P* < .001) (Fig. 3C), and in the multivariate Cox regression analysis, it was 1.040 (95% CI 1.026–1.053) (*P* < .001) (Fig. 4D), indicating that the constructed risk prognostic model for CRlncRNAs could be used as an independent prognostic factor for LUAD.

By applying age, sex, stage, and risk score factors, a nomogram was developed to predict the survival rate of patients with LUAD at 1, 3, and 5 years. If the patient was male (or female), aged 65 years, low-risk group, stage, the total number of points was calculated as 123 according to the corresponding points in the nomogram, and the corresponding 5-year survival rate was 0.724, the 3-year survival rate was 0.868, and the 1-year survival rate was 0.968 (Fig. 4E). The calibration charts for 1, 3, and 5 years were all close to the solid gray line, indicating that the nomogram had good predictive ability (Fig. 4F). The results of the clinical subgroup survival analysis of LUAD showed that the survival of high-risk patients was lower than that of low-risk patients by age (≤ 65 years, >65 years), sex (female, male), and clinical stage (stages I-II and III-IV) (Fig. 4G–L), indicating that this prognostic model applies to patients with different clinical characteristics.

### 3.2. Principal components analysis

**Figure 5.**
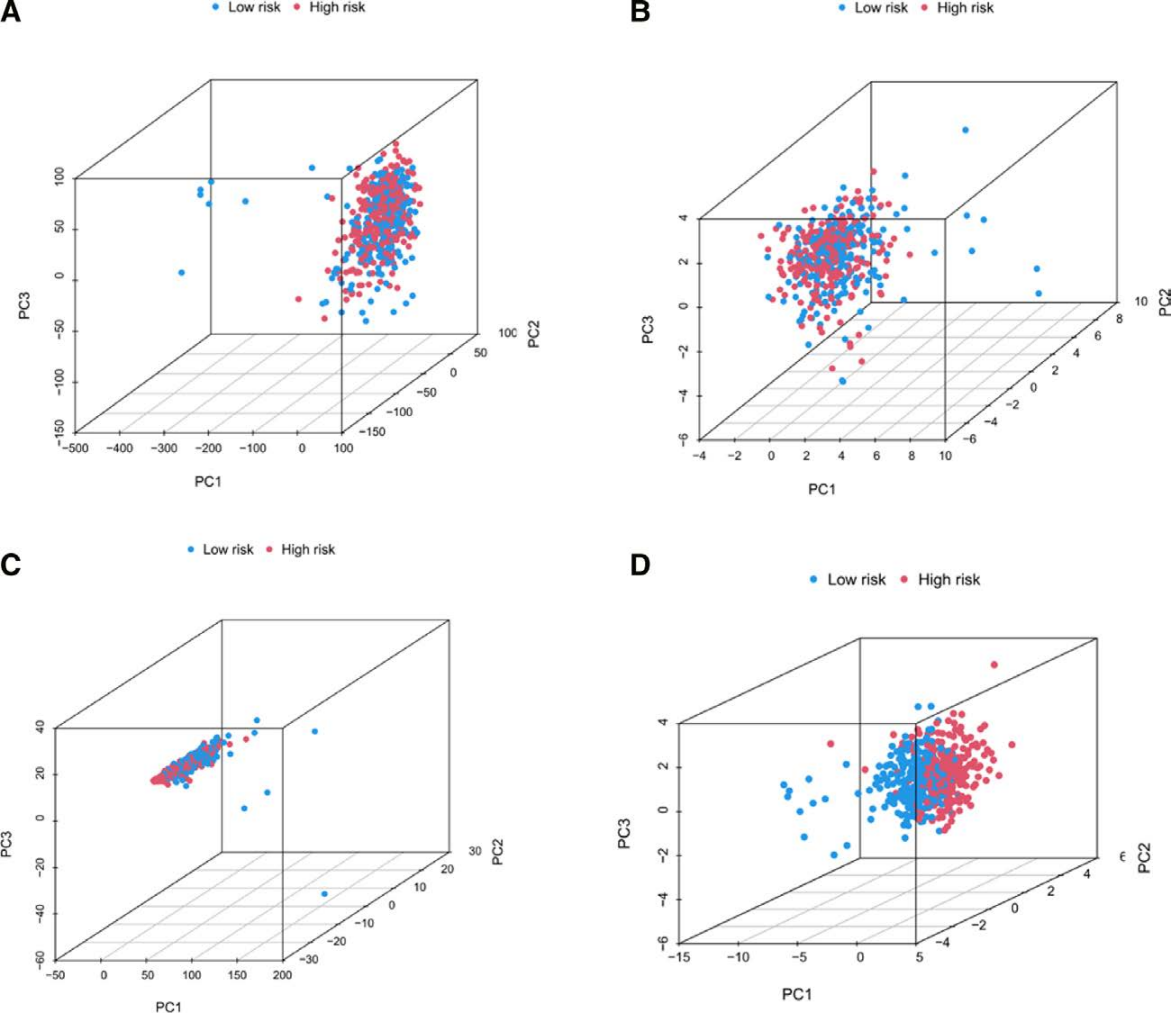
PCA between low and high-risk groups. (A) the all genes set, (B) Cuproptosis Genes set, (C) CRlncRNAs set, (D) 10 CRlncRNAs set. CRlncRNAs = cuproptosis-related lncRNAs, PCA = principal component analysis.

PCA was performed on LUAD samples based on all gene sets, the CRGs set, the CRlncRNA set, and the prognostic model 10-CRlncRNA set. The results showed (Fig. 5) that in the prognostic model 10-CRlncRNA set, the high-risk and low-risk groups were distributed in different distinct directions, suggesting that the CRlncRNA risk model could divide patients with LUAD into 2 groups: high-risk and low-risk, suggesting that the cuproptosis status of the 2 groups was different.

### 3.3. Tumor mutation burden analysis

**Figure 6.**
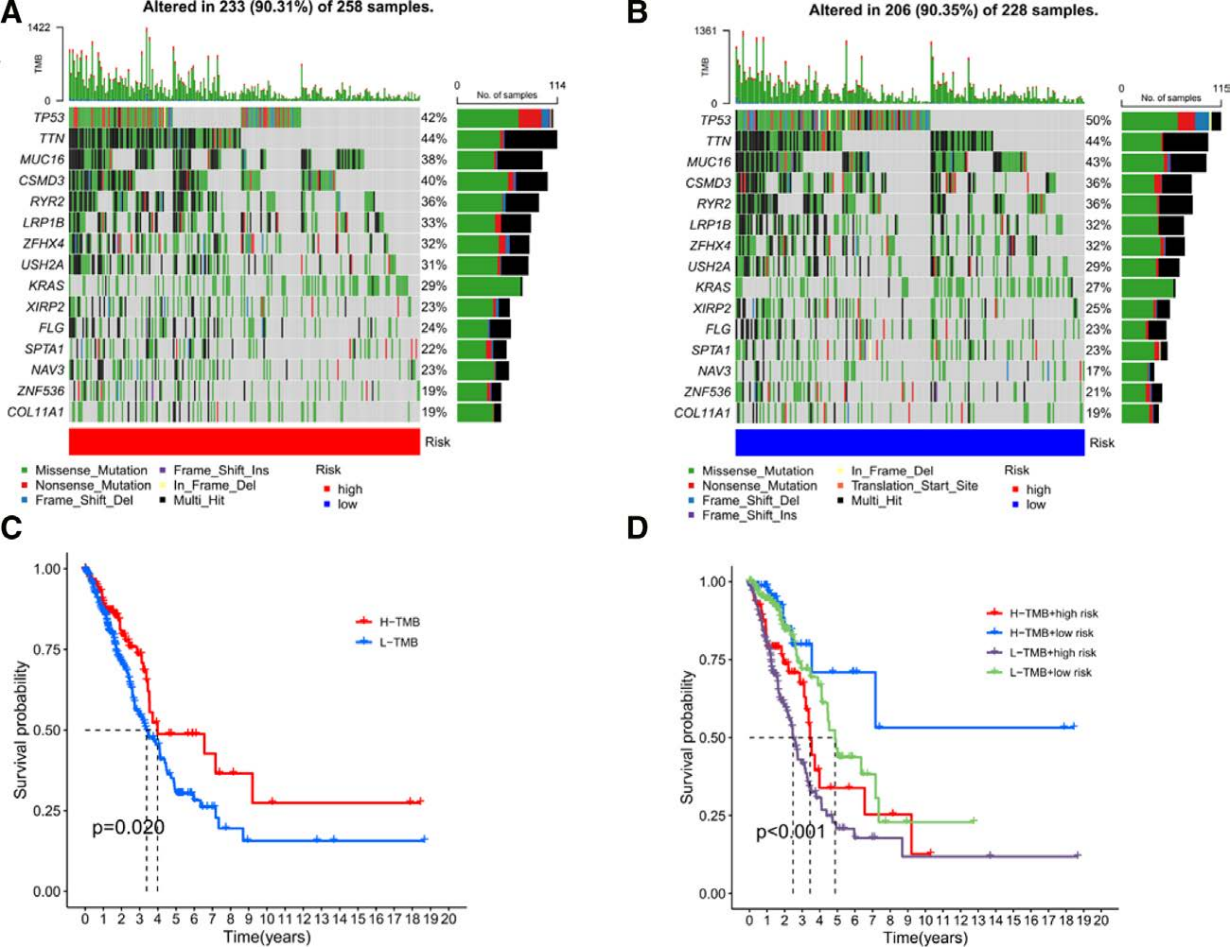
(A–B) Tumor mutation burden analysis of the difference between the high-risk group and low-risk group in LUAD patients; (C) Kaplan–Meier survival analysis of high TMB group (H-TMB) and low TMB group(L-TMB); (D) Kaplan–Meier survival analysis between the groups of H-TMB + high risk, H-TMB + low risk, L-TMB + high risk, and L-TMB + low risk, Comparison of MST between groups: H-TMB + low risk group > L-TMB + low risk group > H-TMB + high risk group > L-TMB + high risk group. LUAD = lung adenocarcinoma, MST = median survival time, TMB = tumor mutational burden.

The TMB of LUAD samples in TCGA database was calculated to analyze the differences in gene mutations between the high- and low-risk groups. The mutation rate in the high-risk group was 90.31%, and the top 5 genes with high mutation frequencies were TTN (44%), TP53 (42%), CSMD3 (40%), MUC16 (38%), and RYR2 (36%) (Fig. 6A). The mutation rate in the low-risk group was 90.35% higher than that in the high-risk group, and the top 5 genes with the highest mutation frequencies were TP53 (50%), TTN (44%), MUC16 (43%), CSMD3 (36%), and RYR2 (36%) (Fig. 6B). Missense mutations were the main mutations in both the LUAD samples. The main form of mutation in both groups of LUAD samples was missense. To investigate the prognostic value of TMB, the samples were divided into high- and low-TMB groups according to the median TMB score, and survival analysis was performed. The results showed that the median survival time (MST) of the high TMB group was better than that of the low TMB group (*P* < .05) (Fig. 6C). Combined survival analysis of TMB and risk score showed a highly statistically significant difference in MST among the 4 groups (*P* < .001), with the best prognosis in the high TMB + low-risk group and the worst prognosis in the low TMB + high-risk group (Fig. 6D).

### 3.4. Chemotherapeutic drugs sensitivity prediction

**Figure 7.**
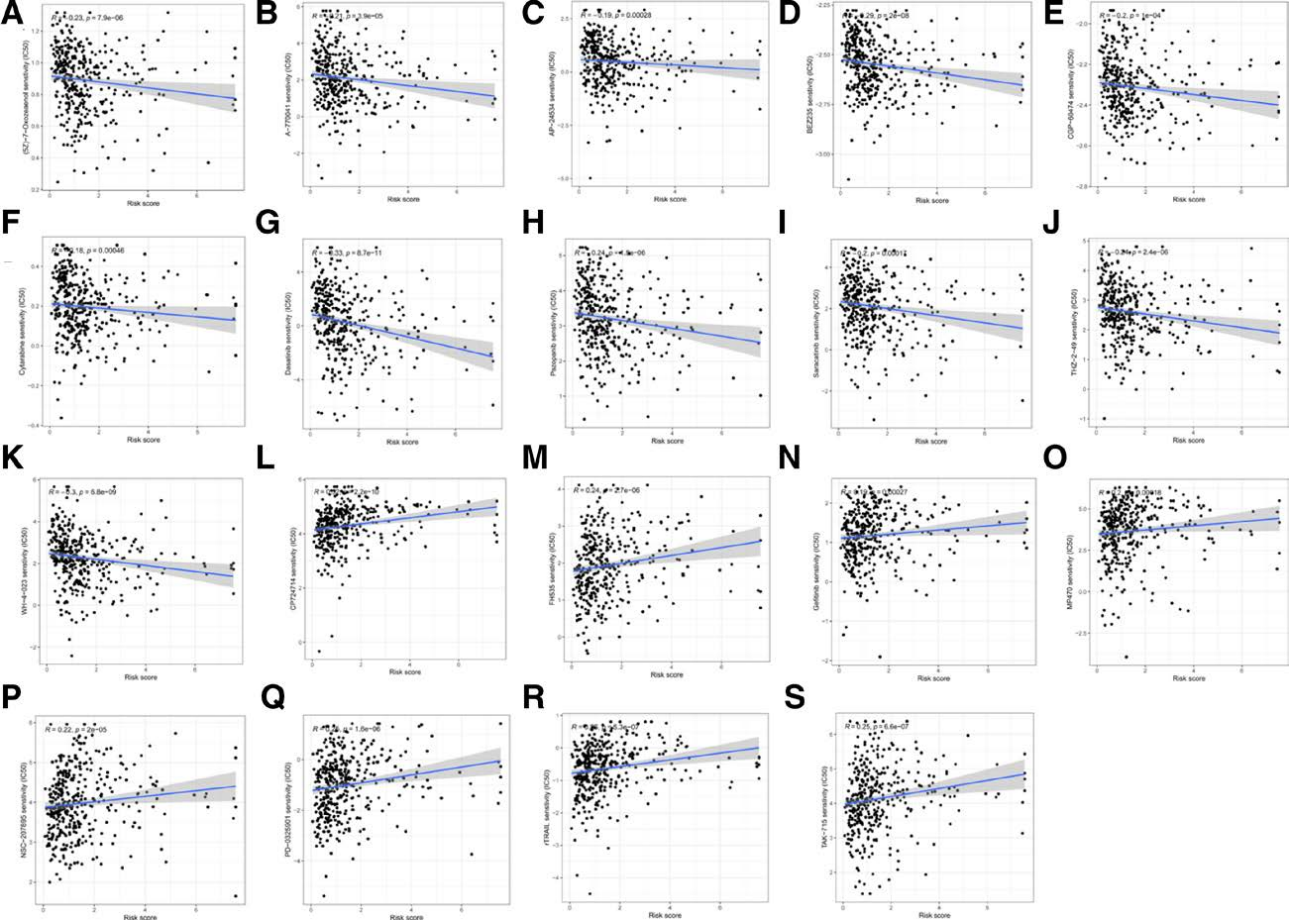
Correlation scatterplot of IC50 of chemotherapeutic drugs and risk score. (A–K) IC50 of chemotherapy drugs was negatively correlated with risk score; (L–S) IC50 of chemotherapy drugs was positively correlated with a risk score. (A) (5Z)-7-Oxozeaenol, (B) A-770041, (C) AP-24534, (D) BEZ235, (E) CGP-60474, (F) Cytarabine, (G) Dasatinib, (H) Pazopanib, (I) Saracatinib, (J) THZ-2-49, (K) WH-4-023, (H) Pazopanib, (I) Saracatinib, (J) THZ-2-49, (K) WH-4-023, (L) CP724714, (M) FH535, (N) Gefitinib, (O) MP470, (P) NSC-207895, (Q) PD-0325901, (R) rTRAIL, (S) TAK-715. IC50 = 50% inhibitory concentration.

**Figure 8.**
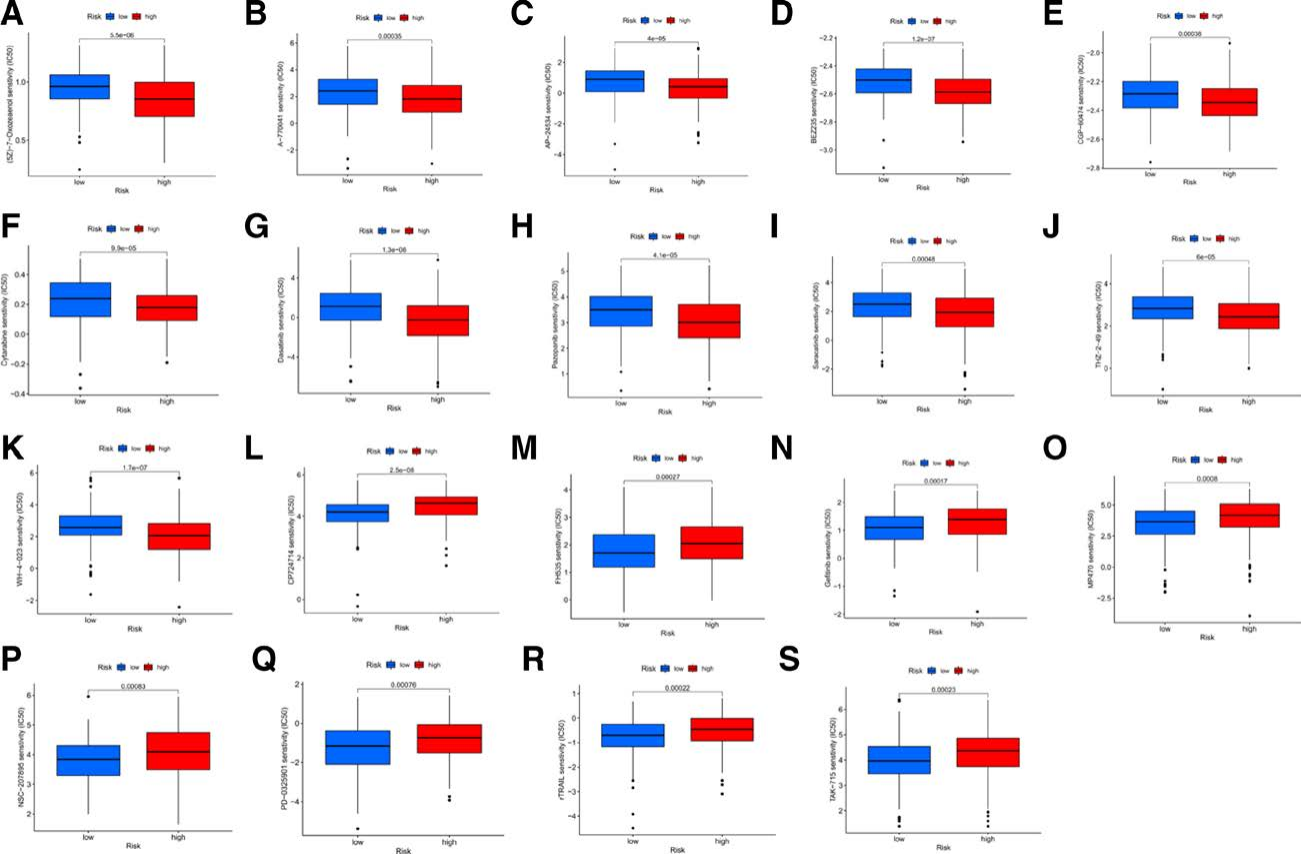
Boxplot showing the mean differences in estimated IC50 of chemotherapy drugs between the high and low-risk groups. (A) (5Z)-7-Oxozeaenol, (B) A-770041, (C) AP-24534, (D) BEZ235, (E) CGP-60474, (F) Cytarabine, (J) Dasatinib, (H) Pazopanib, (I) Saracatinib, (J) THZ-2-49, (K) WH-4-023, (H) Pazopanib, (I) Saracatinib, (J) THZ-2-49, (K) WH-4-023, (L) CP724714, (M) FH535, (N) Gefitinib, (O) MP470, (P) NSC-207895, (Q) PD-0325901, (R) rTRAIL, (S) TAK-715. IC50 = 50% inhibitory concentration.

Chemotherapy is an important treatment option in patients with LUAD. Sensitive chemotherapeutic drugs were screened by predicting their IC50 of chemotherapeutic drugs to further investigate the correlation between the risk and prognosis models and chemotherapeutic drug sensitivity. The results of the study showed that the IC50 values of CP724714, FH535, gefitinib, MP470, NSC-207895, PD-0325901, rTRAIL, and TAK-715, 8 sensitive chemotherapeutic agents, were positively correlated with the risk score (Fig. 7A–H) and were lower in the low-risk group than in the high-risk group (*P* < .001) (Fig. 8A–H), indicating that they could be candidates for LUAD patients in the low-risk group. The IC50 values of (5Z)-7-Oxozeaenol, A-770041, AP-24534, BEZ235, CGP-60474, cytarabine, dasatinib, pazopanib, saracatinib, THZ-2-49, and WH-4-023, 11 chemotherapeutic agents, were negatively correlated with the risk scores were negatively correlated (Fig. 7I–S) and were lower in the high-risk group than in the low-risk group (*P* < .001) (Fig. 8I–S), indicating that they could be used as candidates for LUAD patients in the high-risk group. Therefore, the model risk profile could be used as a potential indicator for predicting drug sensitivity.

## 4. Discussion

LUAD is a highly heterogeneous tumor with complex mechanisms, high morbidity and mortality rates, and a poor prognosis. Robust prognosis prediction models can help accurately assess and predict the prognosis of the disease and formulate accurate individualized treatment strategies. Copper (Cu) is involved in important biological processes related to cancer, including mitochondrial respiration, immune system regulation, antioxidant defense, collagen cross-linking, autophagy, and mitogenic signaling, and plays an important role in cancer.^[24–26]^ Cuproptosis is programmed cell death caused by intracellular copper accumulation that triggers mitochondria. Copper ion carriers have been used as anticancer agents to promote copper death,^[27]^ suggesting that intervention in cuproptosis may be a new target for cancer therapy. LncRNAs play a central role in maintaining various biological activities in tumors and can be used as potential prognostic biomarkers to provide new options for clinical treatment.^[28–30]^ Relevant literatures show that lncRNAs are closely related to the occurrence and development of LUAD,^[31–33]^ and are of great significance for guiding the clinical prognosis of LUAD,^[34–36]^ while the correlation between copper death-related lncRNAs and LUAD needs to be further systematically explored.

Therefore, we constructed a robust and effective prognostic prediction model based on CRlncRNAs to provide new perspectives for diagnosis, treatment, and discovery of new targets for LUAD treatment.

LUAD risk prediction model constructed by Cox analysis, Lasso regression, consisting of 10 CRlncRNAs, including 5 potential high-risk lncRNAs(hazard ratio > 1)(Fig. 2A) MAP3K20-AS1,CRIM1-DT,AC006213.3,AC008035.1,NR2F2-AS1, 5 potentially protective lncRNAs (hazard ratio < 1) (Fig. 2A) AC090948.1, AL356481.1,AC011477.2,AL031600.2, and AC026355.2. Previous studies have shown that MAP3K20-AS1, NR2F2-AS1, AC090948.1, and AC026355.2 are important in the diagnosis, survival prognosis, and clinical treatment of the disease, whereas CRIM1-DT, AC006213.3, AC008035.1, AL356481.1, AC011477.2, AL031600.2 were identified for the first time in disease prognosis prediction.MAP3K20-AS1 is a key molecular marker in the transcriptome sequencing of cardiac fibrosis and heart failure.^[37]^ NR2F2-AS1 can affect the proliferation, invasion, and apoptosis of NSCLC cells by regulating miR-320b targeting BMI1^[38]^ and inducing epithelial-mesenchymal transition in NSCLC by regulating the BVR/ATF-2 pathway through the miR-545-5p/c-Met axis,^[39]^ suggesting that NR2F2-AS1 is a potential therapeutic target for improving NSCLC. AC090948.1 is of great value in predicting the survival of patients with bladder cancer^[40]^ and breast cancer.^[41]^ AC026355.2 is associated with necrotic apoptosis, immunity, and autophagy, and is valuable in predicting LUAD survival as a potential therapeutic target.^[42–44]^ FDX1 is closely related to fatty acid oxidation, amino acid metabolism, and glucose metabolism, which can mediate metabolism and affect prognosis of LUAD.^[45]^ Elesclomol is a highly lipophilic Cu2^+^-binding molecule and FDX1 is a direct target of elesclomol to promote cuproptosis.^[46]^ CRIM1-DT was highly positively correlated with FDX1 (*P* < .001) and AC011477.2 was significantly negatively correlated with FDX1 (*P* < .05), suggesting that both may be closely related to cuproptosis and could be targets for subsequent studies on cuproptosis in LUAD. According to the results of the correlation analysis between CRGs and CRlncRNAs, CRGs and CRlncRNAs with highly significant positive or negative correlation (Fig. 2D) may be potential targets for the subsequent study of LUAD CRlncRNAs.

The model was constructed using a training cohort for OS and mortality prediction, and the results showed poorer OS and higher mortality in the high-risk group than in the low-risk group and were validated in the test cohort and total cohort. The predictive ability of the model for patients with LUAD was verified using clinical multi-index and time-dependent ROC curves. The model was validated as an independent prognostic factor for LUAD using univariate and multinomial Cox regression analysis. A nomogram was constructed using differences in age, sex, staging, and risk scores, and testing was performed by applying calibration charts to enhance the predictive power of the LUAD prognosis. Kaplan–Meier survival analysis by clinical subgroup illustrated that the model is equally applicable to the prediction of survival in patients with different clinical characteristics. In PCA analysis based on the prognostic model of CRlncRNAs, samples were clearly distinguished into high- and low-risk groups, suggesting that cuproptosis status may differ between the 2 groups. This shows that the LUAD prediction model constructed from the above 10 CRlncRNAs has accurate and robust prediction ability.

To better assess the clinical feasibility of the risk model, TMB analysis and chemotherapeutic drug sensitivity prediction were performed. TMB is a key predictor of the clinical benefit of immunotherapy and can predict the survival prognosis of cancer immunotherapy, and related studies have shown that high TMB has a better survival prognosis.^[47,48]^ TP53 mutation is 1 of the common mutations in early LUAD,^[49]^ and both missense and nonsense mutation of TP53 are associated with TMB and elevated neoantigen levels.^[50]^ The TMB results of this study showed that the TP53 mutation rate was in the first place, dominated by missense mutation and nonsense mutation, and the TP53 mutation rate was 50% in the low-risk group, which was higher than 42% in the high-risk group. Survival analysis of samples from the high and low TMB groups showed that MST was better in the high TMB group than in the low TMB group (*P* < .05) (Fig. 7C). Combining TMB with a risk score for survival analysis showed that the high TMB + low-risk group had the best prognosis (*P* < .001) and the low TMB + high-risk group had the worst prognosis (*P* < .001) (Fig. 7D). Thus, this model, combined with TMB, can accurately predict LUAD prognosis and be used as a novel predictive clinical biomarker. In addition, through the correlation analysis between the IC50 of chemotherapeutic drugs and risk score, and the analysis of the difference in sensitivity of chemotherapeutic drugs between high- and low-risk groups, the model can be used as a potential indicator to predict the sensitivity of chemotherapeutic drugs and provide better treatment strategies for patients with LUAD.

However, this study has some limitations. First, this was a retrospective analysis based on publicly available data from the TCGA. Therefore, further prospective analyses are needed to validate the clinical application of CRlncRNA prediction models in patients with LUAD. Second, studies on the mechanism of cuproptosis are still in their initial stages. The number of CRlncRNAs retrieved from the literature is relatively limited, and no studies have yet elucidated the mechanism of lncRNA regulation of cuproptosis in LUAD. This is an exploratory study around the frontiers of science, starting with CRlncRNAs, to open new perspectives for the elucidation of LUAD mechanisms, discovery of potential targets, and clinical treatment.

## Acknowledgments

This study was funded by the Taishan Scholars Construction Project (no. 201712096).

## Author contributions

**Conceptualization:** Huang Di, Jiting Zhao, Zhanjun Qiu, Wei Zhang, Xianhai Chen.

**Data curation:** Huang Di, Yuanlong Hu, Mengjie Wang.

**Formal analysis:** Huang Di, Yuanlong Hu.

**Funding acquisition:** Xue Zhu, Wei Zhang, Xianhai Chen.

**Investigation:** Huang Di, Xue Zhu, Yuanlong Hu, Wei Zhang, Xianhai Chen.

**Methodology:** Huang Di, Jiting Zhao, Xue Zhu, Yuanlong Hu, Zhanjun Qiu, Xianhai Chen.

**Resources:** Xue Zhu, Xinpeng Zhou, Zhanjun Qiu, Xianhai Chen.

**Software:** Huang Di, Xinpeng Zhou, Yuanlong Hu.

**Supervision:** Xue Zhu, Xinpeng Zhou, Zhanjun Qiu.

**Validation:** Wei Zhang.

**Visualization:** Wei Zhang.

**Writing – original draft:** Huang Di, Jiting Zhao, Mengjie Wang, Xianhai Chen.

**Writing – review & editing:** Huang Di, Jiting Zhao, Xinpeng Zhou, Yuanlong Hu, Mengjie Wang, Zhanjun Qiu, Wei Zhang, Xianhai Chen.

## References

[1] Thai AA Solomon BJ Sequist LV. Lung cancer. Lancet. 2021;398:535–54. 34273294 10.1016/S0140-6736(21)00312-3

[2] Travis WD Brambilla E Burke AP. Introduction to The 2015 World Health Organization classification of tumors of the lung, pleura, thymus, and heart. J Thorac Oncol. 2015;10:1240–2. 26291007 10.1097/JTO.0000000000000663

[3] Zappa C Mousa SA. Non-small cell lung cancer: current treatment and future advances. Transl Lung Cancer Res. 2016;5:288–300. 27413711 10.21037/tlcr.2016.06.07 PMC4931124

[4] Xu B Mei J Ji W. LncRNA SNHG3, a potential oncogene in human cancers. Cancer Cell Int. 2020;20:536. 33292213 10.1186/s12935-020-01608-x PMC7640707

[5] Rinn JL Chang HY. Long noncoding RNAs: molecular modalities to organismal functions. Annu Rev Biochem. 2020;89:283–308. 32569523 10.1146/annurev-biochem-062917-012708

[6] Song J Sun Y Cao H. A novel pyroptosis-related lncRNA signature for prognostic prediction in patients with lung adenocarcinoma. Bioengineered. 2021;12:5932–49. 34488540 10.1080/21655979.2021.1972078 PMC8806662

[7] Wu Y Zhang L He S. Identification of immune-related LncRNA for predicting prognosis and immunotherapeutic response in bladder cancer. Aging (Albany NY). 2020;12:23306–25. 33221763 10.18632/aging.104115 PMC7746369

[8] Li X Jin F Li Y. A novel autophagy-related lncRNA prognostic risk model for breast cancer. J Cell Mol Med. 2021;25:4–14. 33216456 10.1111/jcmm.15980 PMC7810925

[9] Tang D Chen X Kroemer G. Cuproptosisa copper-triggered modality of mitochondrial cell death. Cell Res. 2022;32:417–8. 35354936 10.1038/s41422-022-00653-7 PMC9061796

[10] Wang Y Zhang L Zhou F. Cuproptosis:a new form of programmed cell death. Cell Mol Immunol. 2022;19:867–8. 35459854 10.1038/s41423-022-00866-1 PMC9338229

[11] Tsvetkov P Coy S Petrova B. Copper induces cell death by targeting lipoylated TCA cycle proteins. Science. 2022;375:1254–61. 35298263 10.1126/science.abf0529 PMC9273333

[12] Kahlson MA Dixon SJ. Copper-induced cell death. Science. 2022;375:1231–2. 35298241 10.1126/science.abo3959

[13] Deigendesch N Zychlinsky A Meissner F. Copper regulates the canonical NLRP3 Inflammasome. J Immunol. 2018;200:1607–17. 29358279 10.4049/jimmunol.1700712

[14] Tavera-Montañez C Hainer SJ Cangussu D. The classic metal-sensing transcription factor MTF1 promotes myogenesis in response to copper. FASEB J. 2019;33:14556–74. 31690123 10.1096/fj.201901606R PMC6894080

[15] Li Y Hu J Guan F. Copper induces cellular senescence in human glioblastoma multiforme cells through downregulation of Bmi-1. Oncol Rep. 2013;29:1805–10. 23468063 10.3892/or.2013.2333

[16] Liu TT Li R Huo C. Identification of CDK2-related immune forecast model and ceRNA in lung adenocarcinoma, a pan-cancer analysis. Front Cell Dev Biol. 2021;9:682002. 34409029 10.3389/fcell.2021.682002 PMC8366777

[17] Jang JH. Principal component analysis of hybrid functional and vector data. Stat Med. 2021;40:5152–73. 34160848 10.1002/sim.9117 PMC9084921

[18] Samstein RM Lee CH Shoushtari AN. Tumor mutational load predicts survival after immunotherapy across multiple cancer types. Nat Genet. 2019;51:202–6. 30643254 10.1038/s41588-018-0312-8 PMC6365097

[19] Schrock AB Ouyang C Sandhu J. Tumor mutational burden is predictive of response to immune checkpoint inhibitors in MSI-high metastatic colorectal cancer. Ann Oncol. 2019;30:1096–103. 31038663 10.1093/annonc/mdz134

[20] Aykul S Martinez-Hackert E. Determination of half-maximal inhibitory concentration using biosensor-based protein interaction analysis. Anal Biochem. 2016;508:97–103. 27365221 10.1016/j.ab.2016.06.025 PMC4955526

[21] Vis DJ Bombardelli L Lightfoot H. Multilevel models improve precision and speed of IC50 estimates. Pharmacogenomics. 2016;17:691–700. 27180993 10.2217/pgs.16.15 PMC6455999

[22] Geeleher P Cox N Huang RS. pRRophetic: an R package for prediction of clinical chemotherapeutic response from tumor gene expression levels. PLoS One. 2014;9:e107468. 25229481 10.1371/journal.pone.0107468 PMC4167990

[23] Ruli E Ventura L. Accurate likelihood inference for the volume under the ROC surface. Cancer Rep (Hoboken). 2020;3:e1206. 32794638 10.1002/cnr2.1206 PMC7941487

[24] Shanbhag VC Gudekar N Jasmer K. Copper metabolism as a unique vulnerability in cancer. Biochim Biophys Acta Mol Cell Res. 2021;1868:118893. 33091507 10.1016/j.bbamcr.2020.118893 PMC7779655

[25] Oliveri V. Selective targeting of cancer cells by copper ionophores: an overview. Front Mol Biosci. 2022;9:841814. 35309510 10.3389/fmolb.2022.841814 PMC8931543

[26] Lelièvre P Sancey L Coll JL. The multifaceted roles of copper in cancer: a trace metal element with dysregulated metabolism, but also a target or a bullet for therapy. Cancers (Basel). 2020;12:E3594. 10.3390/cancers12123594 PMC7760327 33271772

[27] Ge EJ Bush AI Casini A. Connecting copper and cancer: from transition metal signalling to metalloplasia. Nat Rev Cancer. 2022;22:102–13. 34764459 10.1038/s41568-021-00417-2 PMC8810673

[28] Yang M Lu H Liu J. lncRNAfunc: a knowledgebase of lncRNA function in human cancer. Nucleic Acids Res. 2022;50:D1295–306. 34791419 10.1093/nar/gkab1035 PMC8728133

[29] McCabe EM Rasmussen TP. lncRNA involvement in cancer stem cell function and epithelial-mesenchymal transitions. Semin Cancer Biol. 2021;75:38–48. 33346133 10.1016/j.semcancer.2020.12.012

[30] Tang R Wu Z Rong Z. Ferroptosis-related lncRNA pairs to predict the clinical outcome and molecular characteristics of pancreatic ductal adenocarcinoma. Brief Bioinform. 2022;23:bbab388. 34553745 10.1093/bib/bbab388

[31] Liu Y Liang L Ji L. Potentiated lung adenocarcinoma (LUAD) cell growth, migration, and invasion by lncRNA DARS-AS1 via miR-188-5p/ KLF12 axis. Aging (Albany NY). 2021;13:23376–92. 34644678 10.18632/aging.203632 PMC8544313

[32] Qu S Jiao Z Lu G. PD-L1 lncRNA splice isoform promotes lung adenocarcinoma progression via enhancing c-Myc activity. Genome Biol. 2021;22:104. 33849634 10.1186/s13059-021-02331-0 PMC8042710

[33] Huang W Wang X Wu F. LncRNA LINC00520 aggravates cell proliferation and migration in lung adenocarcinoma via a positive feedback loop. BMC Pulm Med. 2021;21:287. 34496829 10.1186/s12890-021-01657-6 PMC8425021

[34] Xu F Huang X Li Y. m6A-related lncRNAs are potential biomarkers for predicting prognoses and immune responses in patients with LUAD. Mol Ther Nucleic Acids. 2021;24:780–91. 33996259 10.1016/j.omtn.2021.04.003 PMC8094594

[35] Guo Y Qu Z Li D. Identification of a prognostic ferroptosis-related lncRNA signature in the tumor microenvironment of lung adenocarcinoma. Cell Death Discov. 2021;7:190. 34312372 10.1038/s41420-021-00576-z PMC8313561

[36] Lu L Liu LP Zhao QQ. Identification of a ferroptosis-related LncRNA signature as a novel prognosis model for lung adenocarcinoma. Front Oncol. 2021;11:675545. 34249715 10.3389/fonc.2021.675545 PMC8260838

[37] Wang S Lv T Chen Q. Transcriptome sequencing and lncRNA-miRNA-mRNA network construction in cardiac fibrosis and heart failure. Bioengineered. 2022;13:7118–33. 35235759 10.1080/21655979.2022.2045839 PMC8974171

[38] Zhang S Zhang X Sun Q. LncRNA NR2F2-AS1 promotes tumourigenesis through modulating BMI1 expression by targeting miR-320b in non-small cell lung cancer. J Cell Mol Med. 2019;23:2001–11. 30592135 10.1111/jcmm.14102 PMC6378175

[39] Liu C Li QG Zhou Y. LncRNA NR2F2-AS1 induces epithelial-mesenchymal transition of non-small cell lung cancer by modulating BVR/ATF-2 pathway via regulating miR-545-5p/c-Met axis. Am J Cancer Res. 2021;11:4844–65. 34765296 PMC8569365

[40] Zhao K Zhang Q Zeng T. Identification and validation of a prognostic immune-related lncRNA signature in bladder cancer. Transl Androl Urol. 2021;10:1229–40. 33850758 10.21037/tau-20-1353 PMC8039609

[41] Shi GJ Zhou Q Zhu Q. A novel prognostic model associated with the overall survival in patients with breast cancer based on lipid metabolism-related long noncoding RNAs. J Clin Lab Anal. 2022;36:e24384. 35441740 10.1002/jcla.24384 PMC9169174

[42] Gong Z Li Q Li J. A novel signature based on autophagy-related lncRNA for prognostic prediction and candidate drugs for lung adenocarcinoma. Transl Cancer Res. 2022;11:14–28. 35261881 10.21037/tcr-21-1554 PMC8841483

[43] Lu Y Luo X Wang Q. A novel necroptosis-related lncRNA signature predicts the prognosis of lung adenocarcinoma. Front Genet. 2022;13:862741. 35368663 10.3389/fgene.2022.862741 PMC8969905

[44] He C Yin H Zheng J. Identification of immune-associated lncRNAs as a prognostic marker for lung adenocarcinoma. Transl Cancer Res. 2021;10:998–1012. 35116427 10.21037/tcr-20-2827 PMC8798323

[45] Zhang Z Ma Y Guo X. FDX1 can impact the prognosis and mediate the metabolism of lung adenocarcinoma. Front Pharmacol. 2021;12:749134. 34690780 10.3389/fphar.2021.749134 PMC8531531

[46] Tsvetkov P Detappe A Cai K. Mitochondrial metabolism promotes adaptation to proteotoxic stress. Nat Chem Biol. 2019;15:681–9. 31133756 10.1038/s41589-019-0291-9 PMC8183600

[47] Meléndez B Van Campenhout C Rorive S. Methods of measurement for tumor mutational burden in tumor tissue. Transl Lung Cancer Res. 2018;7:661–7. 30505710 10.21037/tlcr.2018.08.02 PMC6249625

[48] Tang B Zhu J Zhao Z. Diagnosis and prognosis models for hepatocellular carcinoma patient’s management based on tumor mutation burden. J Adv Res. 2021;33:153–65. 34603786 10.1016/j.jare.2021.01.018 PMC8463909

[49] Wu C Rao X Lin W. Immune landscape and a promising immune prognostic model associated with TP53 in early-stage lung adenocarcinoma. Cancer Med. 2021;10:806–23. 33314730 10.1002/cam4.3655 PMC7897963

[50] Sun H Liu SY Zhou JY. Specific TP53 subtype as biomarker for immune checkpoint inhibitors in lung adenocarcinoma. EBioMedicine. 2020;60:102990. 32927274 10.1016/j.ebiom.2020.102990 PMC7494676

